# Clinical Application of Estimating Hepatitis B Virus Quasispecies Complexity by Massive Sequencing: Correlation between Natural Evolution and On-Treatment Evolution

**DOI:** 10.1371/journal.pone.0112306

**Published:** 2014-11-13

**Authors:** Maria Homs, Andrea Caballero, Josep Gregori, David Tabernero, Josep Quer, Leonardo Nieto, Rafael Esteban, Maria Buti, Francisco Rodriguez-Frias

**Affiliations:** 1 Centro de investigación biomédica en red: enfermedades hepáticas y digestivas (CIBERehd), Instituto de Salud Carlos III, Barcelona, Spain; 2 Liver Pathology Unit, Departments of Biochemistry and Microbiology, Hospital Vall d'Hebron, Universitat Autònoma de Barcelona, Barcelona, Spain; 3 Virology Unit, Department of Microbiology, Hospital Vall d'Hebron, Universitat Autònoma de Barcelona, Barcelona, Spain; 4 Liver Diseases, Research Institute Hospital Vall d'Hebron, Barcelona, Spain; 5 Liver Unit, Department of Internal Medicine, Hospital Vall d'Hebron, Universitat Autònoma de Barcelona, Barcelona, Spain; University of the Witwatersrand, South Africa

## Abstract

**Aim:**

To evaluate HBV quasispecies (QA) complexity in the preCore/Core regions in relation to HBeAg status, and explore QA changes under natural evolution and nucleoside analogue (NUC) treatment.

**Methods:**

Ultra-deep pyrosequencing of HBV preCore/Core regions in 30 sequential samples (baseline [diagnosis], treatment-free, and treatment-nonresponse) from 10 retrospectively selected patients grouped according to HBeAg status over time: HBeAg+ (N = 4), HBeAg- (N = 2), and fluctuating HBeAg (transient seroreversion/seroconversion pattern) (N = 4). QA complexity was defined by Shannon entropy, mutation frequency, nucleotide diversity, and mutation frequency of amino acids (MfAA) in preCore and Core.

**Results:**

The QA was less complex in HBeAg+ than in HBeAg- or fluctuating HBeAg. High complexity in preCore was associated with decreased viral replication (preCore MfAA negatively correlated with HBV-DNA, p = 0.005). QA complexity in the treatment-free period negatively correlated with values seen during treatment. Specific variants were mainly selected in the Core region in HBeAg- and fluctuating HBeAg patients, suggesting higher immune pressure than in HBeAg+.

**Conclusions:**

The negative correlation between QA natural evolution and on-treatment evolution indicates the importance of pre-treatment QA study to predict QA changes in NUC nonresponders. Study of QA complexity could be useful for managing HBV infection.

## Introduction

Hepatitis B virus (HBV, *Hepadnaviridae* family) causes acute and chronic infection in humans and chimpanzees. Despite the development of successful vaccination programs and effective antiviral therapies, there are more than 240 million carriers of HBV surface antigen (HBsAg) worldwide [1]. Around 150 million of these individuals have active infection and are at a high risk of progressing to cirrhosis or hepatocellular carcinoma [2].

The HBV genome (3.2-kb length) is a partially double-stranded DNA molecule, with four highly overlapping open reading frames: the polymerase, surface, Core, and X [3]. Although the HBV genome has a considerable degree of overlapping (67%) [4], several factors contribute to its significant variability (1.36×10^−3^–6.62×10^−4^ substitutions/site/year) [5]. Some of these are the lack of proofreading of the viral polymerase, the high viral replication rates, and various host enzyme factors, such as guanine to adenine hypermutation activity of the APOBEC3 enzyme [6]. HBV variability yields a swarm of variants that are genetically closely related, but not identical. These evolve, show complex distributions in hosts, and are known as quasispecies (QA) [7]. The HBV QA rebalances its composition to fit to environmental conditions, including host immune stimulation and antiviral treatment [8].

QA study with accurate models and techniques is important to understand the adaptability, pathogenic power, and persistence of HBV, and to optimize strategies to manage and prevent HBV infection [9]. In this sense, Shannon entropy is a useful parameter to study QA complexity in relation to patients' clinical evolution [10], [11]. Various groups have used this approach to study the HBV QA by classic clonal methods, in order to determine the implications of QA complexity on the outcome of infection and the treatment response [12]–[14]. However, the small number of clones analyzed in these studies may significantly bias the Shannon entropy results obtained [15]. Fortunately, a large number of clonal sequences can now be obtained by next-generation technologies, particularly ultra-deep pyrosequencing (UDPS), thereby enabling accurate, quantitative QA description [15]–[17].

Current CHB treatment is mainly based on inhibition of viral polymerase activity by nucleos(t)ide analogues (NUCs), mainly entecavir and tenofovir, both of which have a high genetic barrier, an extremely low probability of resistant variant selection, and considerable suppression of viral replication [18]. However, this antiviral strategy does not affect the HBV intrahepatic reservoir, known as cccDNA. Hence, it is assumed that HBV infection cannot be cured, and the precise duration of treatment remains undefined [18]. Moreover, a significant percentage of patients who previously experienced viral breakthrough after lamivudine, telbivudine, or adefovir treatment are more prone to developing resistance [18].

Several biochemical and virological parameters should be monitored during natural disease evolution and particularly, during antiviral treatment [18]. One of these, HBeAg expression, is associated with a differing course of infection and with the probability of response to antiviral therapy. The presence of HBeAg in serum depends on variants located in the preCore region (main preCore mutation, position 1896) or in the basic core promoter (BCP, positions 1762–1764) [3]. In addition, the Core gene contains epitopic domains that play a central role in the immune response against the virus [19], [20]. Therefore, the preCore/Core is an optimal region to investigate QA evolution in relation to host immune system stimulation. In addition, the preCore/Core regulates HBV replication and includes the only non-overlapping sequence in the HBV genome [3].

The evolution of the preCore/Core regions under NUC treatment has been little investigated. Studies involving molecular cloning of this region have reported that HBeAg seroconversion is associated with increased viral diversity [12], [14]. There is also a recent study in which the preCore/Core was analyzed by UDPS [21] using new bioinformatic tools.

The aim of this study was to evaluate associations between HBeAg status and HBV QA complexity in the preCore/Core region, and to explore QA complexity under natural evolution and under NUC antiviral treatment. To this end, we UDPS-analyzed HBV variability and QA complexity in the preCore/Core region at baseline (at diagnosis of the infection), during a period before starting NUC treatment, and during a period of treatment nonresponse.

## Materials and Methods

### Patients and samples

This is a retrospective study approved by the Ethics Committee of Vall d'Hebron Research Institute, including sequential samples from 10 chronic hepatitis B patients, who gave written consent for participation. The 10 patients were older than 18 years, had detectable HBsAg for more than 6 months, and tested negative for HCV, HIV, and HDV. They were treatment-naïve before starting LVD and failed treatment within two years: 9 patients presented viral breakthrough and one was a primary nonresponder. Furthermore, these patients were selected to include different HBeAg status over time: HBeAg-positive (N = 4), HBeAg-negative (N = 2), and fluctuating HBeAg (ie, a transient seroreversion/seroconversion pattern) (N = 4). Patients with fluctuating HBeAg included 2 patients with seroreversion (HBeAg-negative to positive) and 2 with different periods of seroreversion/seroconversion.

A total of 30 serum samples were UDPS-analyzed. Three samples per patient were analyzed at three time points when HBV DNA was higher than 5 log IU/mL: one at baseline, another after a treatment-free period, and another at treatment nonresponse (Table 1). The baseline (B) sample was the first sample, obtained at the time of the diagnosis. The sample corresponding to the treatment-free (TF) period was one taken after the baseline sample and before starting treatment (median 17 months). The sample at treatment nonresponse (TNR) was subsequent to the treatment-free sample and after treatment nonresponse, with viral breakthrough defined as a confirmed increase in HBV DNA of more than 1 decimal logarithm of IU/mL (log IU/mL) compared to the nadir HBV DNA level on therapy. Primary non-response was defined as less than 1 log IU/ml decrease in HBV DNA level from baseline at 3 months of therapy.

**Table 1:** Description of the patients and samples included in the study.

			Baseline sample				Treatment-free sample				Treatment-nonresponse sample				
Pt	Sex	Year birth	HBV gen	HBV DNA (logIU/mL)	ALT (IU/L)	HBeAg	TF months	HBV DNA (logIU/mL)	ALT (IU/L)	HBeAg	Monthson LVD	HBV DNA (logIU/mL)	ALT (IU/L)	HBeAg	Rescue Treatment
1	M	1958	A	6.1	92	+	13	6.4	75	+	15	>8	84	+	TDF
2	M	1990	D	>8	64	+	18	>8	147	+	13	>8	280	+	TDF+ETV
3	F	1981	D	>8	67	+	11	5.8	60	+	15	5.5	43	+	TDF
4	M	1947	D	7.6	21	+	17	7.2	178	+	48	>8	74	+	TDF
5	M	1943	D	5.5	65	-	15	7.3	117	-	13	5.1	106	-	LVD+TDF
6	M	1980	D	6.2	124	-	32	5.2	200	-	12	>8	210	-	TDF
7*	M	1947	A	7.5	452	+	16	7.5	206	+	17	5.6	125	+	ADV
8	M	1950	D	6	40	+	30	6.6	140	-	13	5.4	43	-	ETV+TDF
9	M	1978	A	>8	83	+	24	5	33	-	33	5.2	18	+	TDF
10	M	1962	A	>8	40	-	18	>8	95	+	15	6.5	65	+	LT

ADV, adefovir; HBV gen, HBV genotype; ETV; entecavir; log IU/mL, decimal logarithm of IU/mL, LVD, lamivudine; TDF, tenofovir; TF, treatment-free; LT, liver transplantation.

**Figure 1:**
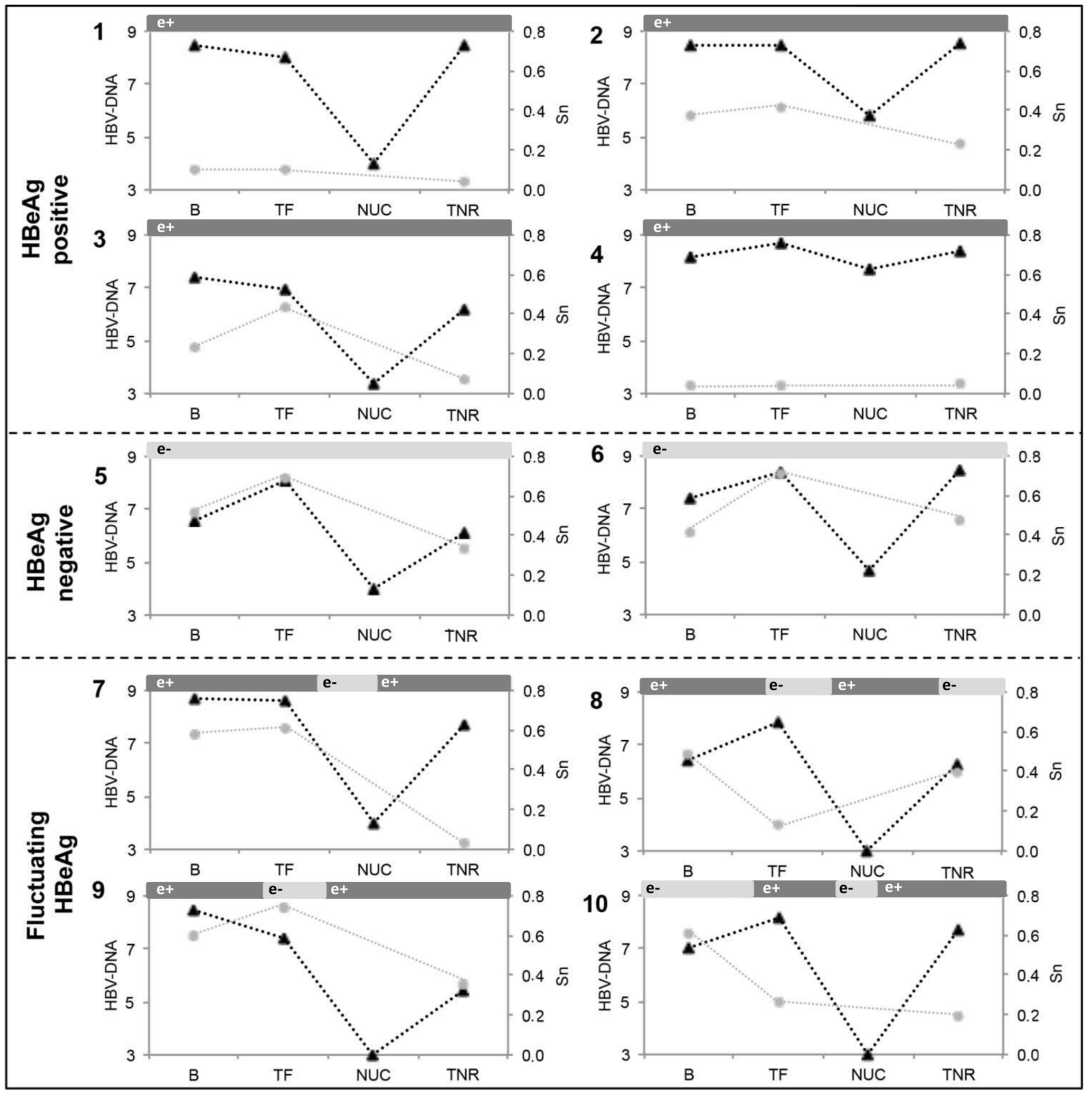
Viral replication and QA evolution of the 10 patients included in the study. The left axis and black-dotted lines represent HBV-DNA (log IU/mL), and the right axis and grey lines represent Sn in baseline (B), treatment-free (TF), and treatment-nonresponse (TNR) samples. HBV DNA level during LVD treatment is indicated as NUC. HBeAg status is shown in bars over the graph: dark grey bar, HBeAg-positive and light grey bar, HBeAg-negative.

*Patient 7 experienced transient HBeAg seroconversion while on treatment, but HBeAg seroreversion occurred before the treatment-nonresponse sample was taken. Seroreversion coincided with virological breakthrough, as is depicted in Figure 1.

The patients' characteristics, the biochemical and virological results for each sample, and the rescue treatment prescribed in each case are presented in Table 1. HBV DNA was quantified with the COBAS Ampliprep/COBAS Taqman HBV Test (Roche Diagnostics). HBV serological markers were determined by commercial immunoassays.

### Amplification and UDPS

The region analyzed by UDPS, clustered positions 1757 to 2152 [16], covered the complete preCore (1814–1900) and the first 84 codons of the Core gene (1901–2152), which include the two main immunodominant epitopes flanked by amino acids 50 to 69 (Th 50–69) and by amino acids 74 to 84 (B74–84) [22].

Briefly, HBV DNA was extracted from 200 µL of serum with the QIAamp DNA MiniKit (QIAGEN, Hilden, Germany). The amplicon library corresponding to the HBV preCore/Core region was obtained after two PCR runs (nested). The first PCR primers were as follows: sense (position 1721–43) 5′ GTTTAAA/GGACTGGGAGGAGC/TTGG 3′ and antisense (position 2804–23) 5′ TGTTCCCAA/GGAATAA/TGGTGA 3′. The nested PCR primers included the recognition site for UDPS, shown in italics. The sequence of the sense primer (position 1737–56) was 5′ *CGTATCGCCTCCCTCGCGCCATCAG*GAGC/TTGGGGGAGGAGAC/TTAG 3′ and the antisense primer (position 2153–80) was 5′*CTATGCGCCTTGCCAGCCCGCTCAG*CCATA/GTTAGTA/GTTA/GACATAAC/TTA/C/GACTAC 3′. To minimize the error rate of the PCR process, high fidelity polymerase (Pfu Ultra-II, Stratagene, La Jolla, USA) was used. The nested PCR products had a length of 494 bp and were isolated from 0.9% agarose gel with the QIAquick extraction Kit (QIAquick Spin Handbook, QIAGEN, Hilden, Germany) and quantified using Quan-iT Picogreen dsDNA reagent (Invitrogen).

Before the UDPS sequencing reaction, each amplicon was pooled to obtain a concentration of 4×10^6^ molecules of the HBV region. This working solution was enriched with the capture beads needed for sequencing. After optimal enrichment, clonal amplification in beads was done in forward and reverse directions (emPCR kits II and III, 454 Life Sciences). UDPS was performed using the Genome Sequencer FLX system (454 Life Sciences).

### UDPS data analysis

FLX 454 data processing was carried out on the open source R environment, using the Biostrings library for pattern matching and sequence alignment, and in-house R scripts [15], [16]. Sequences considered of low quality and those seen in either sense or antisense strands alone were filtered out. Haplotypes found in greater than 0.1% abundance and common to both sense and antisense strands were considered forward and reverse consensus haplotypes (FRCH). Because coverage was not below 10,000 reads, FRCH with a population abundance above 0.25% were considered error-free haplotypes [16]. The HBV genotype of each haplotype was obtained by phylogenetic analysis, using GenBank reference sequences (Table 2).

**Table 2:** NCBI GenBank accession numbers of the reference sequences used in HBV genotyping.

HBV Genotype A	HBV Genotype D
AJ309369	U95551
AJ309370	M32138
AJ344115	X65257
AM282986	X59795
S50225	X02496
V00866	X97848
X02763	X72702
X70185	V01460
Z72479	HE815465
JX507080	

### Parameters for QA complexity

The HBV QA was evaluated including all haplotypes accepted after filter analysis. QA complexity was quantified by three parameters [16]: normalized Shannon entropy (Sn), mutation frequency (Mf), and nucleotide diversity (Pi). Sn measures haplotype diversity attending to the number of haplotypes and their frequency, Mf measures the genetic diversity with respect to the most prevalent haplotype, and Pi measures the population genetic diversity as the average number of mutations per site between each pair of haplotypes in the viral population [15].

In addition, QA complexity was evaluated in terms of the mutation frequency of amino acids in the regions studied. This parameter was calculated separately for the preCore region (48 amino acids) and Core region (84 amino acids).

### Study of QA evolution

To examine QA evolution, two periods were defined: a treatment-free period (median 17 months), and a period under treatment pressure (median 15 months). The differences found for each parameter (Sn, Mf, and Pi) between the B and TF samples were considered measures of natural evolution. Differences between the TF and TNR samples were considered measures of evolution occurring under treatment pressure.

### Statistical analysis

All data are presented as the median and range. Statistical analyses were carried out using IBM SPSS 20 (SPSS Inc., Chicago, USA). Correlation analyses were performed with Spearman's rho test. The Mann-Whitney *U* test and Kruskal-Wallis test were used for comparisons of two independent variables. Significance was set at p≤0.05.

In a previous study we demonstrated the applicability of the *t* or *Z* test in statistical inference with indices of viral quasispecies diversity [15]. In the present study, we used the more stringent Spearman rho correlation test for this purpose, in keeping with the analyses used in recent studies on QA evolution under antiviral treatment and the treatment response [12], [13].

## Results

Thirty samples from 10 patients were analyzed (3 samples/patient at 3 time points: B, TF, and TNR). After applying the quality filters (FRCH above 0.25%) 986,014 sequences were analyzed, and high coverage per patient was obtained (median 98,494 sequences per patient, range 59,039–136,660). UDPS sequencing data have been submitted to the GenBank SRA database (BioProject accession number PRJNA260562, BioSample accession numbers in Table 3). HBV DNA, alanine aminotransferase (ALT) levels, and HBeAg status of each sample are presented in Table 1. All changes detected in the 30 samples are presented in Table S1 and the dominant haplotypes of all samples are aligned in File S1. HBV genotype of the 10 patients, which is also indicated in Table 1, showed no changes over the sequential study.

**Table 3:** Biosample accession numbers for each sample analyzed.

Patient	Sample	Biosample accession number
1	Baseline	SAMN03023656
1	Treatment-free	SAMN03023657
1	Treatment nonresponse	SAMN03023658
2	Baseline	SAMN03023659
2	Treatment-free	SAMN03023660
2	Treatment nonresponse	SAMN03023661
3	Baseline	SAMN03023662
3	Treatment-free	SAMN03023663
3	Treatment nonresponse	SAMN03023664
4	Baseline	SAMN03023665
4	Treatment-free	SAMN03023666
4	Treatment nonresponse	SAMN03023667
5	Baseline	SAMN03023668
5	Treatment-free	SAMN03023669
5	Treatment nonresponse	SAMN03023670
6	Baseline	SAMN03023671
6	Treatment-free	SAMN03023672
6	Treatment nonresponse	SAMN03023673
7	Baseline	SAMN03023674
7	Treatment-free	SAMN03023675
7	Treatment nonresponse	SAMN03023676
8	Baseline	SAMN03023677
8	Treatment-free	SAMN03023678
8	Treatment nonresponse	SAMN03023679
9	Baseline	SAMN03023680
9	Treatment-free	SAMN03023681
9	Treatment nonresponse	SAMN03023682
10	Baseline	SAMN03023683
10	Treatment-free	SAMN03023684
10	Treatment nonresponse	SAMN03023685

Overall, QA complexity in all samples yielded the following median values: Sn 0.366 (range, 0.026–0.738), Mf 7.7×10^−4^ (range, 0.7×10^−5^–1.7×10^−2^), and Pi 1.43×10^−3^ (range, 1.3×10^−5^–2.8×10^−2^). Significant correlations were found between the three variables used to study HBV QA complexity (Rs _Sn-MF_ 0.914, p<0.001; Rs _Sn-Pi_ 0.910, p<0.001; and Rs _Sn-MF_ 0.998, p<0.001), indicating that the three approaches provided very similar estimations. In the overall population, median preCore mutation frequency of amino acids (MfAA) was 2.827 (range, 0–284.35) and median Core MfAA was 6.682 (0–399.92).

### QA complexity and its relationship with sample time points, ALT levels, HBV genotype, HBV DNA quantification, and HBeAg status

The QA distribution, HBV DNA level, and HBeAg status of each patient are depicted in Figure 1. To simplify presentation of the results, the QA is represented by Sn. In each patient, the distribution of Mf and Pi was the same as that of Sn, but at a smaller scale (data not shown), as all parameters significantly correlated. Analysis of differences in QA complexity between B, TF, and TNR samples showed significantly higher Sn (and greater diversity) in B (0.45, range 0.0367–0.607) compared to TNR (0.21, range 0.026–0.478) (p = 0.035), nonsignificantly higher values in TF (0.42, range, 0.045–0.738) relative to TNR (0.21, range 0.026–0.478) (p = 0.075), and similar values between B and TF samples.

There were no significant differences in any complexity parameter (Sn, Pi or Mf) between genotypes A and D, nor were there significant correlations between these parameters and HBV DNA or ALT levels. On separate analysis of the preCore and Core regions, preCore MfAA showed a significant negative correlation with HBV DNA level (Rs preCoreMfAA-DNA −0.5, p = 0.005), whereas MfAA values in Core increased as HBV DNA decreased, but a significant correlation was not found. Keeping in mind the small sample size, these findings suggest that increased preCore complexity is associated with decreased HBV viral replication. To avoid possible effects of treatment, only B and TF samples were analyzed. No significant differences in QA complexity parameters were observed between HBV genotypes, and complexity did not correlate with HBV DNA or ALT levels.

On assessment of QA complexity and HBeAg status in the 30 samples, we found significantly lower QA complexity in HBeAg-positive patients than in HBeAg-negative ones, as determined by Sn, Mf, and Pi, and by MfAA in preCore and Core (Table 4). To explore differences in QA complexity according to HBeAg positive or negative status, and to avoid possible effects of treatment on HBeAg, only B and TF samples were analyzed. As was seen in the total of samples, Sn and Pi results showed greater complexity in HBeAg-negative than positive samples (Sn, p = 0.03; Pi, p = 0.037, Table 4). preCore- and Core-MfAA did not significantly differ between HBeAg-positive and negative samples.

**Table 4:** HBV QA complexity attending to HBeAg status in the 30 samples analyzed and in the 20 without treatment pressure.

		All samples N = 30			Samples without treatment N = 20		
QA parameter		HBeAg (+)	HBeAg (−)	p	HBeAg (+)	HBeAg (−)	p
**Sn**	Median	0.23	0.49	0.003	0.35	0.42	0.03
	Range	(0.03–0.61)	(0.13–0.74)		(0–1.7×10^−2^)	(0.04–0.74)	
**Mf**	Median	2.8×10^−4^	2.8×10^−3^	0.003	7.4×10^−4^	1.35×10^−3^	NS
	Range	(0–1.1×10^−2^)	(5.4×10^−4^–1.7×10^−2^)		(0–1.2×10^−3^)	(0–1.7×10^−2^)	
**Pi**	Median	5.3×10^−4^	4.0×10^−3^	0.002	1.3×10^−4^	2.37×10^−3^	0.037
	Range	(0–1.4×10^2^)	(10^3^–2.8×10^2^)		(0–1.7×10^−4^)	(0–2.8×10^−5^)	
**pcMfAA**	Median	1.41	44	0.024	1.67	51.75	NS
	Range	(0–192.06)	(0–284)		(0–192.1)	(0–197)	
**CoreMfAA**	Median	1.35	45.74	0.011	1.32	24.15	NS
	Range	(0–399.94)	(0.95–207.2)		(0.34–399.9)	(0.95–164.42)	

### QA complexity and HBeAg status: longitudinal study

**Figure 2:**
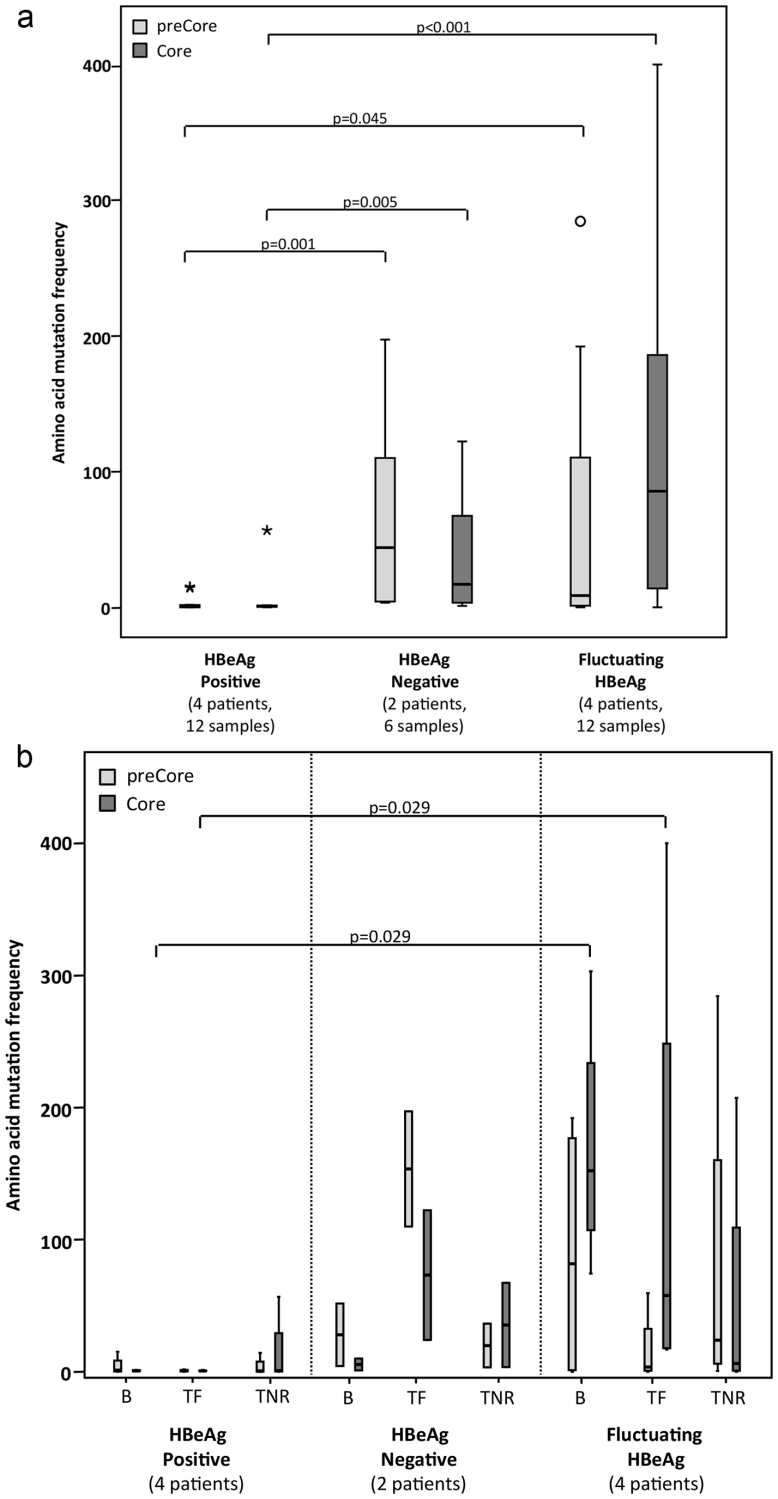
Distribution of mutated amino acids in the preCore and Core regions (a) in patients grouped by HBeAg evolution and (b) splitting into HBeAg evolution and the three time points: baseline (B), treatment-free (TF) and treatment-nonresponse (TNR).

To explore possible associations between HBeAg status and QA variability over time, patients were divided into three groups: the HBeAg+ group included patients with persistently positive HBeAg (cases 1–4); the HBeAg- group, patients with persistently negative HBeAg (cases 5 and 6), and HBeAg+/−, patients with fluctuating HBeAg (cases 7–10) (Figure 1). Sn, Mf, and Pi results for each sample are presented in Table S2. QA complexity was significantly lower in HBeAg+ than HBeAg- patients (Sn p = 0.001, Mf p = 0.002, and Pi p = 0.002) or the HBeAg+/− group (Sn p = 0.014, Mf p = 0.004, and Pi p = 0.003). There were no significant differences between HBeAg- and HBeAg+/− patients. In an attempt to define whether the variability was located in the preCore or Core region, MfAA was calculated for these regions separately in each HBeAg status group (Figure 2a). preCore and Core MfAA were both significantly lower in HBeAg+ than in HBeAg- (preCore MfAA, p = 0.001 and Core MfAA, p = 0.005) or HBeAg+/− (preCore MfAA, p = 0.045 and Core MfAA, p = 0.001) patients, and there were no differences between the HBeAg- and HBeAg+/− groups.

To explore possible differences in QA complexity between the sampling time points, parameters were calculated according to HBeAg status over time and the sample analyzed (B, TF, and TNR). QA complexity was systematically higher in HBeAg- and HBeAg+/− than in HBeAg+ at all three time points (Table 5). However, statistically significant differences in Sn, Mf, and Pi were only found at baseline between HBeAg+ and HBeAg+/− patients (p = 0.029). preCore and Core MfAA results at the three time points are depicted in Figure 2b. Interestingly, both parameters indicated significantly greater complexity in HBeAg+/− cases than HBeAg+ ones in B and TF samples (p = 0.029). With regard to the lack of significance in HBeAg- cases, it should be remembered that only two patients always tested HBeAg-negative.

**Table 5:** Shannon entropy (Sn), mutation frequency (Mf), and nucleotide diversity (Pi) at three time points in patients grouped by HBeAg evolution.

		HBeAg-Positive (N = 4)			HBeAg-Negative (N = 2)			Fluctuating HBeAg (N = 4)		
		Baseline*	TF	TNR	Baseline	TF	TNR	Baseline*	TF	TNR
**Sn**	Median	0.1664	0.2597	0.06165	0.4695	0.69735	0.40545	0.58545	0.43615	0.2778
	Range	0.037–0.374	0.045–0.431	0.044–0.230	0.420–0.519	0.685–0.710	0.333–0.478	0.48–0.61	0.128–0.74	0.026–0.39
**Mf**	Median	1.95×10^−4^	4.11×10^−4^	3.70×10^−5^	1.3×10^−3^	4.63 ×10^−3^	1.44 ×10^−3^	5.61 ×10^−3^	1.49 ×10^−3^	5.16 ×10^−4^
	Range	0.15–10×10^−4^	0.19–22.3×10^−4^	1.9–26×10^−5^	1.27–1.35×10^−3^	3.22–6.05×10^−3^	0.55–2.33×10^−3^	1.65–11.7×10^−3^	0.68–10×10^−3^	0.06–1710×10^−4^
**Pi**	Median	3.81×10^−4^	6.24E-04	7.35×10^−5^	1.31×10^−3^	6.55×10^−3^	2.23×10^−3^	7×10^−3^	2.37×10^−3^	9.96×10^−4^
	Range	0.3–10×10^−4^	0.38–35×10^−4^	3.7–4.8×10^−5^	1.27–2.43×10^−3^	4.74–8.36×10^−3^	1.05–3.42×10^−3^	2.37–14.7×10^−3^	1.33–17×10^−3^	0.1–282×10^−4^

*Significant differences (p = 0.029) between HBeAg-positive and fluctuating HBeAg patients in baseline samples.

TF, treatment-free; TNR, treatment-nonresponse.

### Distribution of preCore and Core changes

In 5 patients (cases 2, 3, 5, 7, and 10), the nucleotide changes encountered were mainly located in the Core region, 4 patients (cases 1, 4, 8, and 9) showed changes in both the preCore and Core, and in the last patient (case 6), changes were mainly located in the preCore region. No specific patterns of variability were observed at the different time points (B, TF, and TNR).

The detection of preCore variants was specific for each patient and each sample (Table S1), but we have summarized the main mutations responsible for HBeAg expression (BCP and preCore mutations) (Table 6). These particular mutations were more frequent in the HBeAg+/− group (patients 7, 8, 9 and 10) than in HBeAg+ or HBeAg- patients.

**Table 6:** Percentage of main preCore variants in the samples analyzed.

			preCore variants	
Pt	Sample	HBeAg status	BCP	main preCore variants
**1**	B	+	wt	wt
	TF	+	wt	wt
	TNR	+	wt	wt
**2**	B	+	wt	wt
	TF	+	wt	wt
	TNR	+	wt	wt
**3**	B	+	A1762T/G1764A (0.27%)	wt
	TF	+	wt	wt
	TNR	+	wt	wt
**4**	B	+	wt	wt
	TF	+	wt	wt
	TNR	+	wt	wt
**5**	B	-	A1762T/G1764A (100%)	G1896A/G1899A (100%)
	TF	-	A1762T/G1764A (100%)	G1896A/G1899A (100%)
	TNR	-	A1762T/G1764A (100%)	G1896A/G1899A (100%)
**6**	B	-	A1762T/G1764A (100%)	G1896A (89.1%)/G1899A (10.92%)
	TF	-	A1762T/G1764A (100%)	G1896A (73.1%)/G1899A (26.9%)
	TNR	-	A1762T/G1764A (100%)	G1896A (100%)
**7**	B	+	A1762T/G1764A (99.44%)	wt
	TF	+	A1762T/G1764A (100%)	wt
	TNR	+	A1762T/G1764A (100%)	wt
**8**	B	+	A1762T/G1764A (100%)	G1896A (38.23%)/G1899A (58.83%)
	TF	-	A1762T/G1764A (100%)	G1896A (98%)/G1899A (0.77%)
	TNR	-	A1762T/G1764A (80.41%)	G1896A (80.41%)
**9**	B	+	A1762T/G1764A (100%)	G1899A (0.34%)
	TF	-	A1762T/G1764A (100%)	G1896A (11.64%)/G1899A (0.49%)
	TNR	+	A1762T/G1764A (100%)	wt
**10**	B	-	A1762T/G1764A (100%)	wt
	TF	+	A1762T/G1764A (100%)	wt
	TNR	+	A1762T/G1764A (100%)	wt

BCP, Basal core promoter; wt, wild-type: samples that showed 100% of wild-type main BCP or preCore variants.

Five cases (patients 1, 2, 4, 5, and 7; Table S1) showed a slight tendency to accumulate variability in Core epitopic regions (Th50–69, nucleotide positions 2048–2107, and B74–84, positions 2120–2152), in agreement with the role of Core as an immune-stimulating region. However, positive selection of Core variants in the HBV QA, defined as selection of a new master sequence that differed from the master sequence in the previous sample, was more frequent in HBeAg- (2 of 2) and HBeAg+/− (4 of 4) patients than in HBeAg+ patients (1 of 4) (Table 7), a finding that may suggest enhancement of the host immune response due to the lack of HBeAg.

**Table 7:** Patients with Core positive selection. Cases in which the new variant was detected in previous samples as minor variants are also indicated.

Pt	Sample	Core positive selection	Minor variant	Sample
**3**	TNR	V63, 100%	-	-
**5**	TNR	E83, 100%	D83E	TF (13.97%)
**6**	TF*	S12, 73.1% - Q79, 60.52%	-	-
**7**	TF	S79, 54.15%	P79S	B (30.63%)
	TNR	G63-M66-P79, 100%	V63M-T66M-S79P	B (032%) and TF (0.47%)
**8**	TF*	Q14-A35-H57-V58-T59-D64-N67, 98%	-	-
**9**	TF	H5-I60, 88.36%	P5H-L60I	B (47.39%)
	TNR	P5-L60, 98.09%	H5P-I60L	TF (11.64%)
**10**	TF*	E40, 98.03% -S41, 92.29%	D40E-A41S	B (0.32)

*Detected in the treatment-free (TF) and maintained in the treatment-nonresponse (TNR) sample.

### Evolution of QA complexity without treatment and under NUC pressure

**Figure 3:**
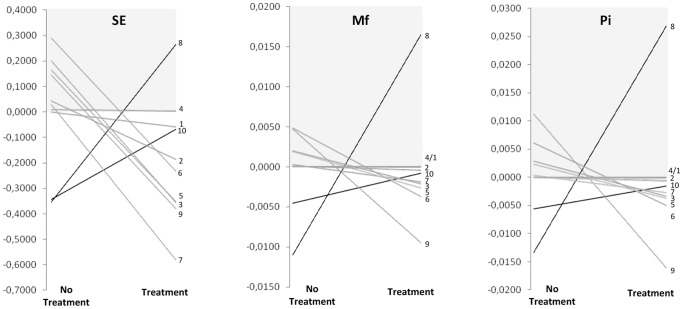
Patterns of QA complexity in the period without and with treatment, according to Sn, Mf, and Pi. The vertical axis represents differences between the complexity parameter (Sn, Mf or Pi) during the time period indicated in horizontal axis, No Treatment (TF-B) and Treatment (TNR-TF). The grey areas highlight positive values and represent an increase in HBV QA complexity.

QA evolution in each of the 10 patients in the TF and TNR periods is shown in Figure 3. In most cases (7/10), there was an increase in QA complexity during natural evolution (grey areas in Figure 3) and a decrease in QA complexity under treatment. One case (patient 4) showed no changes in QA complexity between TF and TNR using any of the three parameters, and two patients, both HBeAg+/− (cases 8 and 10), showed an inverse behavior: a decrease in QA complexity without treatment and an increase during treatment (black lines, Figure 3).

Despite the different patterns observed among the 10 patients, QA complexity at TF showed a significant negative correlation with the complexity at TNR regarding Sn (Rs −0.661, p = 0.038), Mf (Rs −0.891, p = 0.01) and Pi (Rs −0.903, p<0.001), suggesting that the evolution of preCore/Core QA complexity during the time without treatment determines the evolution of these regions during treatment.

## Discussion

The HBV QA composition and the changes that occur over QA evolution are important factors related to controlling and treating chronic HBV infection. Hence, acquiring accurate knowledge of HBV QA complexity is currently a major challenge for managing chronic hepatitis B patients. Recent reports support the concept that QA complexity is a clinically relevant factor in the course and prognosis of this disease and in the response to treatment [12]–[14], [23]. In this line, HBV QA complexity has been associated with the antiviral response in ETV-treated patients, in whom lower complexity was seen in responders than in partial responders [13]. Cheng et al. [12], [14] reported higher viral diversity in HBeAg natural or treatment-induced (IFN or LVD) seroconverters than in non-seroconverters, thereby providing evidence that increased viral diversity is associated with HBeAg seroconversion, in agreement with our observations in the present study. Although these studies [12], [14] have provided valuable findings, the techniques used analyzed only small numbers of clones, and the results may not be representative of the overall viral population, which contains billions of particles. In contrast, next-generation sequencing methods, particularly UDPS used in the present study, enable clonal analysis of thousands of sequences in a single sample [8], [24]–[29], provide a number of clonal sequences to lend reliability to calculation of QA complexity parameters [15], and have robust quantitative and reproducibility values, making them viable alternatives to molecular cloning for QA study [16].

In this study, we applied UDPS to determine QA complexity in the HBV preCore and Core regions. More than 19,500 sequences per sample were analyzed, a number considerably higher than the numbers in recent reports on the HBV QA (mean 20–26 clones/sample) [12]–[14], [23] and clearly sufficient to guarantee QA calculations [15]. To determine HBV QA complexity, three parameters were used: Shannon entropy (Sn), mutation frequency (Mf), and nucleotide diversity (Pi). The high correlation between the results obtained with these parameters indicates that Sn, Mf and Pi equally represented HBV QA complexity. In addition, MfAA was used to independently explore variability at the amino acid level in both the preCore and Core regions.

We found significant differences in the QA complexity parameters according to HBeAg status, with greater complexity in HBeAg- than HBeAg+ samples, in agreement with previous studies [12], [14]. However, the higher complexity (Sn, Mf and Pi) seen in HBeAg- cases lacked significance when analyzing QA complexity attending to HBeAg evolution, likely because of the small number of HBeAg-negative patients. Interestingly, both the HBeAg- and HBeAg+/− groups showed significantly higher QA complexity than HBeAg+ patients when complexity was analyzed at the amino acid level (MfAA) in the preCore and Core regions separately. Therefore, our data provide evidence that increased viral diversity is associated with HBeAg seroconversion and strongly suggest significant evolutionary enhancement that was even more evident in fluctuating HBeAg status. These findings may indicate an increase in evolutionary pressure due to a more intense immune response in HBeAg-negative status, likely associated with the lack of HBeAg and its immunomodulatory effect [30].

Overall, there were no significant differences in QA complexity between A and D genotypes in the 30 samples analyzed, despite the constraints on main preCore mutation selection and HBV genotype [31]. Moreover, no correlation was observed between ALT levels and QA variability. Although it is assumed that ALT status provides an estimate of the strength of the immunological response against viral infection [32], ALT can be influenced by many factors and a single point measurement may not be indicative of the long-term immune status of a host [32]. Furthermore, the aim of this study was to sequentially analyze HBV QA complexity with deep clonal sequence coverage to guarantee the complexity calculations, and because of the huge amount of data involved, only ten patients were included.

The negative correlation between HBV DNA levels and QA complexity in the present study agrees with recent findings [12], [14]. Although the correlations did not achieve statistical significance, the three main QA complexity parameters (Sn, Mf and Pi) showed that the higher the HBV DNA level, the lower was QA complexity in preCore/Core. However, it should be remembered that all samples included had a high viral load (HBV DNA >5 log IU/mL). This potentially confounding factor may have contributed to the absence of significance in the correlation studies. Nonetheless, in the separate analysis of the preCore and Core regions at the amino acid level (MfAA calculation), the significant negative correlation with HBV DNA observed in preCore MfAA suggests that the preCore mutated variants could confer a decrease in preCore fitness to regulate HBV replication. The preCore contains the essential encapsidation signal for viral replication, and substitutions in this region may interfere with its functionality [3].

The Sn in preCore/Core was significantly higher at B than at TNR, and was non-significantly higher in TF than at TNR. These findings suggest a decrease in QA evolution, associated with LVD treatment failure. Boni et al [33] reported that the immune response is enhanced when antiviral treatment controls HBV replication, but our patients were treatment nonresponders and therefore in a situation contrary to that of Boni's population [33]. In fact, most of our cases showed a decrease in QA complexity at TNR, which likely reflects attenuation of immune system activity at nonresponse.

The possible relationship between QA complexity and antiviral therapy response was not tested in this study because responders, who have undetectable HBV-DNA levels after treatment, were not included. However, longitudinal UDPS study of our patients enabled examination of evolutionary patterns in the absence and presence of antiviral treatment, and this yielded conclusive results according to HBeAg status. Some patterns were identified: at baseline, QA complexity in HBeAg-positive patients was significantly lower than in those with fluctuating HBeAg (Table 5). In addition, HBeAg-positive patients showed lower Core gene variability (Core MfAA) than those with fluctuating HBeAg at baseline and TF. In contrast, preCore MfAA was not significantly different between any of the groups, which could indicate that the host immune response mainly acts against Core epitopes in patients with fluctuating HBeAg. In fact, preCore variants and positive selection of Core variants were common in HBeAg-negative and fluctuating patients, in keeping with results from our previous studies [8], [22]. These findings may result from HBV adaptation under host immune pressure, or even be due to an effect of antiviral treatment on the Core gene.

HBV QA evolution was analyzed in two periods, natural evolution and under NUC pressure. The significant negative correlation in QA complexity between the two periods suggests that changes occurring in natural evolution might be affected by the host immune response and determine the evolution of the same region under NUC treatment. We found that in most patients, the greater the complexity during natural evolution, the more homogeneous was the population after treatment, indicating that NUCs might also have some indirect effects on the preCore/Core region. However, this pattern was not observed in 2 of the 10 patients studied, probably due to the transient serconversion/seroreversion status of these cases (fluctuating HBeAg). Apart from these differences, the main inverse pattern of QA evolution in the two periods may indicate the following: under immune pressure, a complex population evolves, but during antiviral therapy pressure, a limited number of HBV variants carrying resistant mutations emerge and predominate, rendering the QA less complex in a type of bottleneck phenomenon.

The main limitation of this study is the small sample of 10 patients sequentially analyzed, which was related to our aim to characterize the HBV QA as accurately as possible by very high coverage (median, 98,494 sequences per patient). All cases were longitudinally studied, and 3 samples per patient were included to enable examination of evolutionary patterns in the absence and presence of antiviral treatment. Thus, sample size was mainly determined by the huge amount of data to process and the cost of UDPS.

In conclusion, the results of this study provide further evidence of the utility of UDPS for investigating the evolution of the HBV QA. In addition, they provide confirmatory data for previous findings in studies with lower analytical coverage indicating greater QA variability in HBeAg-negative than HBeAg-positive patients. Our results show that high complexity in the preCore region is associated with low viral replication, in keeping with the key role of this region in HBV replication, and suggest an enhanced immune response in HBeAg-negative patients, probably related to the lack of HBeAg immunomodulatory activity. In the same direction, the positive selection of Core variants in HBeAg-negative and fluctuating status can be understood as a potential mechanism to escape the host immune system by nucleocapsid sequence changes. Finally, the strong negative correlation of QA evolution in the treatment-free period and under treatment shows the importance of studying the QA before treating patients, as a potential predictive factor of HBV evolution in cases of NUC nonresponse. With the consolidation of next-generation sequencing methods that enable the reproduction of viral haplotype study, QA complexity parameters could be useful for clinical management of HBV infection.

## Supporting Information

Table S1 Percentages of changes observed in baseline (B), treatment-free (TF) and treatment non-response (TNR) sample of each patient. Nucleotide changes are registered in relation to the dominant haplotype of each sample.
(PDF)

Table S2 Normalized Shannon Entropy (Sn), mutation frequency (Mf) and nucleotide diversity (Pi), and mutation frequency of amino acids in the preCore (pre Core MfAA) and Core (Core MfAA) regions for each sample.
(PDF)

File S1 Alignment of the dominant haplotypes of the 30 samples analyzed.
(FAS)
