# Supplementary material for: Clinical Application of Estimating Hepatitis B Virus Quasispecies Complexity by Massive Sequencing: Correlation between Natural Evolution and On-Treatment Evolution
Source: PLoS One. 2014 Nov 13;9(11):e112306. doi: 10.1371/journal.pone.0112306 (PMC4231103; doi:10.1371/journal.pone.0112306)
Supplement: Table S1 — Percentages of changes observed in baseline (B), treatment-free (TF) and treatment non-response (TNR) sample of each patient. Nucleotide changes are registered in relation to the dominant haplotype of each sample. (PDF) [file pone.0112306.s001.pdf]

Supplementary table 1. Percentages of changes observed in baseline (B), treatmet-free (TF) and treatment non-response (TNR) sample of each patient. Nucleotide changes are registered in relation to the dominant haplotype of each sample.

Patient	Sample	eAg	Change	%	Change	%	Change	%	Change	%	Change	%	Change	%	Change	%
1	B	+	**C1788T**	0.459	**A1827C**	0.357	R39Q	0.949	E64K	0.255						
	TF	+	**C1788T**	0.629	**A1827C**	0.284	R39Q	0.264	E64K	0.284						
	TNR	+														
2	B	+	E64K	0.453	P79Q	0.252										
	TF	+	E64K	0.442												
	TNR	+	E64K	0.446	P79Q	0.821										
3	B	+	**T1759C**	4449	**A1761C**	2.570	**A1762T**	0.274	**G1764A**	0.274	**T1767A**	2.153	**T1767G**	0.274	**T1771A**	0.874
	TF	+	**C1802T**	0.317	**T1822C**	0.317	P20S	0.450	L60P	0.705						
	TNR	+	**A1757G**	0.958	**T1759C**	0.887	**A1761C**	0.534	**T1767A**	0.353	**A1775G**	4.093	**C1802T**	0.958	**G1803T**	0.958
4	B	+	**A1827C**	0.271	E64K	0.321										
	TF	+	**A1827C**	0.362	E64K	0.392										
	TNR	+	**A1757G**	0.562	E64K	0.261										
5	B	-	**A1768T**	0.438	**C1799T**	0.5311	**C1810A**	0.458	**T1825C**	1.281	**A1827C**	0.937	**T1878A**	0.750	A11V	1.645
	TF	-	**C1766T**	1.913	P5A	0.264	S44A	0.972	A54T	0.317	L76V	0.285	A80P	1.163	S81A	3.477
	TNR	-	**C1773T**	8.059	**G1787A**	0.298	**C1802T**	0.452	**G1803A**	0.596	**G1809A**	4.163	**C1810T**	0.308	**A1838T**	2.889
6	B	-	**C1802T**	0.252	**A1867C**	2.999	**A1896G**	10.915	**G1899A**	10.915	P20S	0.263	A34T	0.252	A54T	0.303
	TF	-	**A1896G**	26.9	**G1899A**	26.9	S12T	26.9	V74A	39.474	Q79P	39.474				
	TNR	-	**C1802T**	0.424	**G1803A**	0.403	**G1809A**	0.448	**C1882T**	0.413	**G1891A**	0.403	P20S	0.496	A36V	0.372
7	B	+	**T1762A**	0.556	**A1764G**	0.556	V63G	0.326	T66M	0.708	T67S	1.8	N74T	29.514	P79S	31.223
	TF	+	A58D	2.074	G63V	2.547	T66M	3.343	T67S	1.724	N74T	28.204	S79P	36.099	P79L	0.473
	TNR	+	**G1809A**	0.253												
8	B	+	**G1809A**	0.435	**C1810T**	0.912	**A1827C**	0.383	**A1846T**	28.925	**G1896A**	38.230	**A1899G**	41.173	E14Q	37.805
	TF	-	**T1846A**	2.268	**A1896G**	1.999	**G1899A**	0.766	Q14E	1.998	A35S	1.999	H57Q	2.288	V58A	2.288
	TNR	-	**G1757A**	2.311	**T1762A**	19.594	**A1764G**	19.594	**C1773T**	17.283	**A1775G**	1.035	**G1803A**	0.382	**A1808T**	0.703
9	B	+	**T1766C**	47.913	**G1803A**	0.491	**A1850T**	46.659	**C1882T**	0.282	**G1891A**	0.282	**G1899A**	0.335	P5H	48.301
	TF	-	**T1773C**	11.643	**G1787A**	0.943	**G1803A**	1.339	**G1809A**	2.282	**T1846A**	11.643	**C1858T**	11.643	**C1869T**	0.619
	TNR	+	**G1780A**	0.303	**G1787A**	0.253	**C1802T**	0.2629	**G1803A**	0.717	**A1808G**	1.689	**G1809A**	0.404	**A1850T**	9.626
10	B	-	Y38H	1.092	E40Q	39.495	E40D	39.325	A41S	19.344	I59T	0.403	I59V	42.454	G63V	0.308
	TF	+	**C1802T**	0.270	**A1850T**	0.591	T12S	0.830	E40D	1.970	A41S	7.705	I59V	3.412	G63V	0.539
	TNR	+	**A1775G**	0.715	**C1802T**	0.322	**G1809T**	0.806	**T1822A**	0.534	**T1823C**	0.282	**T1824C**	1.723	**C1828A**	0.554

Supplementary table 1.

Patient	Sample	eAg	Change	%	Change	%	Change	%	Change	%	Change	%	Change	%	Change	%
1	B	+														
	TF	+														
	TNR	+														
2	B	+														
	TF	+														
	TNR	+														
3	B	+	**A1772C**	0.437	**C1788T**	0.253	**C1802T**	0.284	P20S	0.295						
	TF	+														
	TNR	+	**T1846A**	0.958	**C1882T**	0.362	T12S	0.958	V27I	0.958	D40E	0.958	V63G	42.872	T67N	0.958
4	B	+														
	TF	+														
	TNR	+														
5	B	-	A44T	0.604	A44S	0.604	H47Q	0.312	S81A	2.968	D83E	2.624				
	TF	-	D83E	13.973												
	TNR	-	**G1891A**	0.832	D2G	0.617	P20S	0.606	A34T	0.257	A54T	0.545	A54V	0.411	Q77Z	0.298
6	B	-														
	TF	-														
	TNR	-	E40D	0.786	A54T	0.434	V74A	29.165	Q79P	27.935						
7	B	+	P79L	0.698												
	TF	+														
	TNR	+														
8	B	+	S35A	37.640	Q57H	37.267	A58V	37.267	I59T	37.267	E64D	37.267	T67N	36.935	V74I	5.45
	TF	-	T59I	2.288	D64N	1.543	D64E	1.543	N67T	2.289	V74I	0.332				
	TNR	-	**A1809G**	19.951	**C1810T**	2.311	**A1811T**	2.311	**T1846A**	2.312	**T1850A**	17.283	**T1858C**	17.280	**A1859G**	16.499
9	B	+	Y6H	0.586	F9Y	0.335	L15Z	0.272	P20S	0.440	R28Z	0.565	L31I	1.161	S44T	23.56
	TF	-	**G1896A**	11.643	**G1899A**	0.487	H5P	11.643	F9Y	4.270	T12S	4.270	E14Q	15.33	D22H	1.927
	TNR	+	**C1851T**	1.891	**C1869T**	0.344	**C1882T**	0.546	**G1891A**	0.354	P5H	1.911	P20S	0.95	S21A	1.911
10	B	-	T67N	4.02	T70S	0.944	L84Q	0.838								
	TF	+	L84Q	0.383												
	TNR	+	**A1850T**	0.877	**T1871A**	0.393	W71L	0.393	D83H	0.494						

Supplementary table 1.

Patient	Sample	eAg	Change	%	Change	%	Change	%	Change	%	Change	%	Change	%	Change	%
1	B	+														
	TF	+														
	TNR	+														
2	B	+														
	TF	+														
	TNR	+														
3	B	+														
	TF	+														
	TNR	+	V74S	0.958	D83E	0.958										
4	B	+														
	TF	+														
	TNR	+														
5	B	-														
	TF	-														
	TNR	-	Q77E	0.298												
6	B	-														
	TF	-														
	TNR	-														
7	B	+														
	TF	+														
	TNR	+														
8	B	+														
	TF	-														
	TNR	-	**C1882T**	0.432	**G1891A**	0.261	**A1896G**	19.594	Q14E	19.594	L16P	16.499	F24L	0.412	T33I	5.295
9	B	+	C48Z	0.356	A54T	0.293	L60I	47.391	A69G	0.314						
	TF	-	E40Q	11.643	S44T	5.152	A54T	0.426	A54V	0.396	I60L	11.643	A69V	0.436	N74A	11.643
	TNR	+	A34T	0.445	C48Y	0.465	A54T	0.677	A58V	0.334	L60I	1.911	G73S	0.293	G73D	0.324
10	B	-														
	TF	+														
	TNR	+														

Supplementary table 1.

Patient	Sample	eAg	Change	%	Change	%	Change	%	Change	%	Change	%	Change	%	Change	%
1	B	+														
	TF	+														
	TNR	+														
2	B	+														
	TF	+														
	TNR	+														
3	B	+														
	TF	+														
	TNR	+														
4	B	+														
	TF	+														
	TNR	+														
5	B	-														
	TF	-														
	TNR	-														
6	B	-														
	TF	-														
	TNR	-														
7	B	+														
	TF	+														
	TNR	+														
8	B	+														
	TF	-														
	TNR	-	A35S	19.594	H57Q	19.594	V58A	19.594	T59I	19.594	D64E	19.303	N67T	19.595	V74N	17.283
9	B	+														
	TF	-	P79Q	4.696												
	TNR	+	E77K	0.283												
10	B	-														
	TF	+														
	TNR	+														
