# Supplementary material for: Clinical Application of Estimating Hepatitis B Virus Quasispecies Complexity by Massive Sequencing: Correlation between Natural Evolution and On-Treatment Evolution
Source: PLoS One. 2014 Nov 13;9(11):e112306. doi: 10.1371/journal.pone.0112306 (PMC4231103; doi:10.1371/journal.pone.0112306)
Supplement: Table S2 — Normalized Shannon Entropy (Sn), mutation frequency (Mf) and nucleotide diversity (Pi), and mutation frequency of amino acids in the preCore (pre Core MfAA) and Core (Core MfAA) regions for each sample. (PDF) [file pone.0112306.s002.pdf]

Pt	Condition	Sn	Mf	Pi	preCore MfAA	Core Mfaa
1	B	0,1	8,30E-05	1,65E-04	1,67	1,39
	TF	0,1	8,40E-05	1,67E-04	1,88	0,64
	TNR	0,04	1,90E-05	3,70E-05	0,00	0,00
2	B	0,37	6,61E-04	1,03E-03	0,00	0,82
	TF	0,42	7,38E-04	1,08E-03	0,00	0,52
	TNR	0,23	2,60E-04	4,79E-04	0,00	1,47
3	B	0,23	3,07E-04	5,96E-04	15,17	0,34
	TF	0,43	2,23E-03	3,50E-03	0,65	1,33
	TNR	0,08	5,32E-05	1,06E-04	14,25	56,73
4	B	0,04	1,50E-05	3,00E-05	0,56	0,38
	TF	0,05	1,91E-05	3,80E-05	0,75	0,46
	TNR	0,05	2,08E-05	4,10E-05	1,17	0,31
5	B	0,52	1,35E-03	2,43E-03	4,29	9,89
	TF	0,68	3,22E-03	4,74E-03	197,10	24,15
	TNR	0,33	5,46E-04	1,05E-03	36,29	3,47
6	B	0,42	1,27E-03	2,26E-03	51,75	0,95
	TF	0,71	6,05E-03	8,36E-03	110,00	122,21
	TNR	0,48	2,33E-03	3,42E-03	3,38	67,33
7	B	0,58	1,65E-03	2,37E-03	2,28	74,36
	TF	0,61	1,89E-03	2,71E-03	0,00	399,92
	TNR	0,03	6,60E-06	1,30E-05	0,54	0,00
8	B	0,48	1,17E-02	1,47E-02	161,31	303,07
	TF	0,13	6,79E-04	1,33E-03	5,56	18,82
	TNR	0,39	1,71E-02	2,82E-02	284,35	207,19
9	B	0,59	5,59E-03	6,33E-03	192,06	140,07
	TF	0,74	1,03E-02	1,76E-02	59,48	96,82
	TNR	0,36	8,03E-04	1,54E-03	35,96	11,06
10	B	0,61	5,63E-03	7,67E-03	0,00	164,42
	TF	0,26	1,09E-03	2,04E-03	1,73	16,84
	TNR	0,2	2,29E-04	4,55E-04	11,73	1,49
